# Effects of thermal conditioning on the performance of *Pocillopora acuta* adult coral colonies and their offspring

**DOI:** 10.1007/s00338-021-02123-9

**Published:** 2021-07-21

**Authors:** Crystal J. McRae, Wen-Bin Huang, Tung-Yung Fan, Isabelle M. Côté

**Affiliations:** 1 Department of Natural Resources and Environmental Studies, National Dong Hwa University, Hualien, Taiwan; 2 Department of Biological Sciences, Simon Fraser University, British Columbia, Canada; 3 Institute of Marine Biology, National Dong Hwa University, Pingtung, Taiwan; 4 National Museum of Marine Biology and Aquarium, Pingtung, Taiwan

**Keywords:** Thermal pre-conditioning, Acclimation, Resilience, Climate change, Taiwan

## Abstract

Ocean warming induced by climate change is the greatest threat to the persistence of coral reefs globally. Given the current rate of ocean warming, there may not be sufficient time for natural acclimation or adaptation by corals. This urgency has led to the exploration of active management techniques aimed at enhancing thermal tolerance in corals. Here, we test the capacity for transgenerational acclimation in the reef-building coral *Pocillopora acuta* as a means of increasing offspring performance in warmer waters. We exposed coral colonies from a reef influenced by intermittent upwelling and constant warm-water effluent from a nuclear power plant to temperatures that matched (26 °C) or exceeded (29.5 °C) season-specific mean temperatures for three reproductive cycles; offspring were allowed to settle and grow at both temperatures. Heated colonies reproduced significantly earlier in the lunar cycle and produced fewer and smaller planulae. Recruitment was lower at the heated recruitment temperature regardless of parent treatment. Recruit survival did not differ based on parent or recruitment temperature. Recruits from heated parents were smaller and had lower maximum quantum yield (Fv/Fm), a measurement of symbiont photochemical performance. We found no direct evidence that thermal conditioning of adult *P. acuta* corals improves offspring performance in warmer water; however, chronic exposure of parent colonies to warmer temperatures at the source reef site may have limited transgenerational acclimation capacity. The extent to which coral response to this active management approach might vary across species and sites remains unclear and merits further investigation.

**Supplementary Information:**

The online version contains supplementary material available at 10.1007/s00338-021-02123-9.

## Introduction

Coral reefs have declined dramatically over the past several decades (Bellwood et al. 2004; Bruno and Selig 2007; Hughes et al. 2017a). This is of concern because coral reefs harbour high biodiversity (Côté and Knowlton 2013) and provide critical ecosystem services to millions of people globally (Woodhead et al. 2019). The many drivers of coral decline include a range of natural (e.g. disease, storms) and human-induced (e.g. pollution, fishing) local stressors (Nyström et al. 2000; Burke et al. 2011). But it is chronic ocean warming and acute thermal anomalies induced by climate change that pose the greatest threat to the persistence of corals (Hoegh-Guldberg et al. 2007, 2017; Carpenter et al. 2008; Hughes et al. 2017b).

Corals are sensitive to warming; even small increases in ocean temperature can result in substantial physiological changes in these habitat-forming species. For example, increased water temperature can alter reproductive timing (Crowder et al. 2014; Fan et al. 2017), fertilization/embryogenesis (Negri et al. 2007; Albright and Mason 2013), larval physiology (Putnam et al. 2010; Edmunds et al. 2011), pelagic dispersal (Heyward and Negri 2010; Figueiredo et al. 2014), growth (Edmunds 2008; Cantin et al. 2010) and survival of corals (Randall and Szmant 2009; McManus et al. 2019). Mean global sea surface temperature has already risen by 1 °C since pre-industrial levels, mostly in the past 50 years and additional warming of ~ 1–4 °C is expected by 2100 (IPCC 2019). A temperature increase of this nature will surpass the physiological thermal limits of many coral species, leading to coral bleaching and mortality (Hoegh-Guldberg 1999; Frieler et al. 2013). Mass coral bleaching events, which have occurred episodically at a global scale since 1998 (Wilkinson 1998), are now occurring with increased frequency and magnitude (Hughes et al. 2018; Eakin et al. 2019; IPCC 2019).

The extent to which climate change-induced warming will affect corals in the short term rests primarily on the corals’ capacity to withstand, and potentially adapt to, warming conditions. Coral thermal tolerance is highly variable (Fitt et al. 2001). There are a wide range of temperature thresholds observed across species and reef sites (Loya et al. 2001; Oliver et al. 2011; van Woesik et al. 2011), and the mechanisms and duration of these tolerances are diverse and dynamic (Brown et al. 2014; Carballo-Bolaños et al. 2020). For example, corals from warmer (Howells et al. 2016a, b) or thermally variable reefs (Safaie et al. 2018) and species possessing specific traits (e.g. thick tissues; Putnam et al. 2017) or a high capacity for heterotrophic feeding (Grottoli et al. 2006) can show higher resistance and/or resilience to higher temperatures. Tolerance, however, is not static; coral species that were ‘winners’ in the past can become ‘losers’ during subsequent heat stress events (Grottoli et al. 2014) and corals thought to be thermally sensitive can acclimate relatively quickly (within ~ 2 years) when translocated to a thermally variable reef (Palumbi et al. 2014). This fluidity in thermal tolerance provides a glimmer of hope for corals: a high plasticity that might lead to acclimation or adaptation to a warming ocean. By better understanding the mechanisms underlying plasticity in thermal tolerance, scientists and managers may be able to harness this capacity and actively enhance coral resistance and resilience.

One proposed management approach focused on coral thermal tolerance is assisted evolution (van Oppen et al. 2015). Since the current rate of ocean warming is greater than what corals have experienced in the past (Pandolfi et al. 2011), there is concern that corals will not be able to adapt quickly enough to match the pace of environmental change (Hoegh-Guldberg et al. 2007; Matz et al. 2018; van Oppen et al. 2017). Assisted evolution aims to give corals a ‘helping hand’ by speeding up natural acclimation and adaptation processes, thereby actively increasing coral resistance and resilience to ocean warming (van Oppen et al. 2015). One potential means of accomplishing this is through thermal preconditioning of adult coral colonies to trigger an epigenetic response that could increase the thermal tolerance of their offspring—a process variably referred to as transgenerational acclimation (van Oppen et al. 2015), transgenerational transfer (Ho and Burggren 2010), transgenerational plasticity (Torda et al. 2017; Donelson et al. 2018), or cross- and multigenerational plasticity (Bryne et al. 2020). Although a wide range of organisms have been the focus of transgenerational acclimation studies (Ho and Burggren 2010; Bryne et al. 2020), corals remain relatively understudied. Putnam and Gates (2015) first showed that preconditioning of adult coral colonies to increased temperature and pCO_2_ could lead to metabolic acclimation in offspring. Subsequently, there have been few studies that explore the potential of this new technique as a means of mitigating coral decline in the face of climate change (but see Bellworthy et al. 2019; Putnam et al. 2020). Likely explanations for the paucity of transgenerational acclimation studies on corals include the temporal, logistical and financial challenges of assessing multi-generational effects on relatively long-lived species. Simplifying the process of transgenerational acclimation might therefore provide a useful tool both to assess the extent of benefits to corals and to implement it for management purposes.

Here, we used a relatively simple, low-tech approach to assess the outcomes of transgenerational acclimation, which considers ecological rather than molecular or genetic metrics of ‘success’. Specifically, we asked whether thermal preconditioning of adult colonies of the reef-building coral *Pocillopora acuta* provides a benefit to offspring when the latter are subsequently exposed to high temperature. *P. acuta* is a hermaphroditic species with a mixed, yet predominately brooding, reproductive strategy, with larvae produced both asexually and sexually (Yeoh and Dai 2010; Smith et al. 2019). We examined the influence of adult conditioning across three critical coral life-history stages: reproduction, recruitment and recruit performance, and assessed ecological metrics that are easily measured such as larval abundance, survival, size and symbiont photochemical performance.

## Materials and methods

### Study site and coral collection

We collected 24 colonies of *P. acuta* (diameter, mean ± SD: 13.56 ± 2.2 cm) (Kenting National Park collection permit #: 1,050,002,277) in February 2017 from depths of 3–5 m at Outlet Reef, a fringing reef in Nanwan Bay, southern Taiwan (Fig. S1). Outlet Reef is situated near Taiwan’s 3rd nuclear power plant and, consequently, experiences summer temperatures that are typically ~ 2–3 °C warmer than the surrounding reefs in the region (Keshavmurthy et al. 2012). Despite widespread bleaching after the construction and initial operation of the nuclear plant in the late 1980s (Hung et al. 1998), corals in the area have recovered, albeit with a change in community composition (Keshavmurthy et al. 2014). In addition to the warming influence of the nuclear plant effluent, Outlet Reef can experience substantial fluctuations in daily temperature (typically ± 2–3 °C, but a drop of up to 10 °C has been recorded) due to internal tide-induced upwelling in Nanwan Bay (Lee et al. 1997; Jan et al. 2004).

Collected coral colonies were immediately transported to the research center at the nearby National Museum of Marine Biology and Aquarium (NMMBA) and held in a recovery tank for 7 days where they were monitored for signs of bleaching and disease. Colonies were held in a semi-enclosed outdoor area exposed to ambient light, partly shaded to approximate conditions at the collection site; water temperature (25.1 °C ± 0.7) was similar to that experienced at Outlet reef (25.4 °C ± 0.5 in February 2017). Seawater in the flow-through recovery system was sand-filtered and sourced offshore from NMMBA.

### Experimental system and parental treatments

**Fig. 1:**
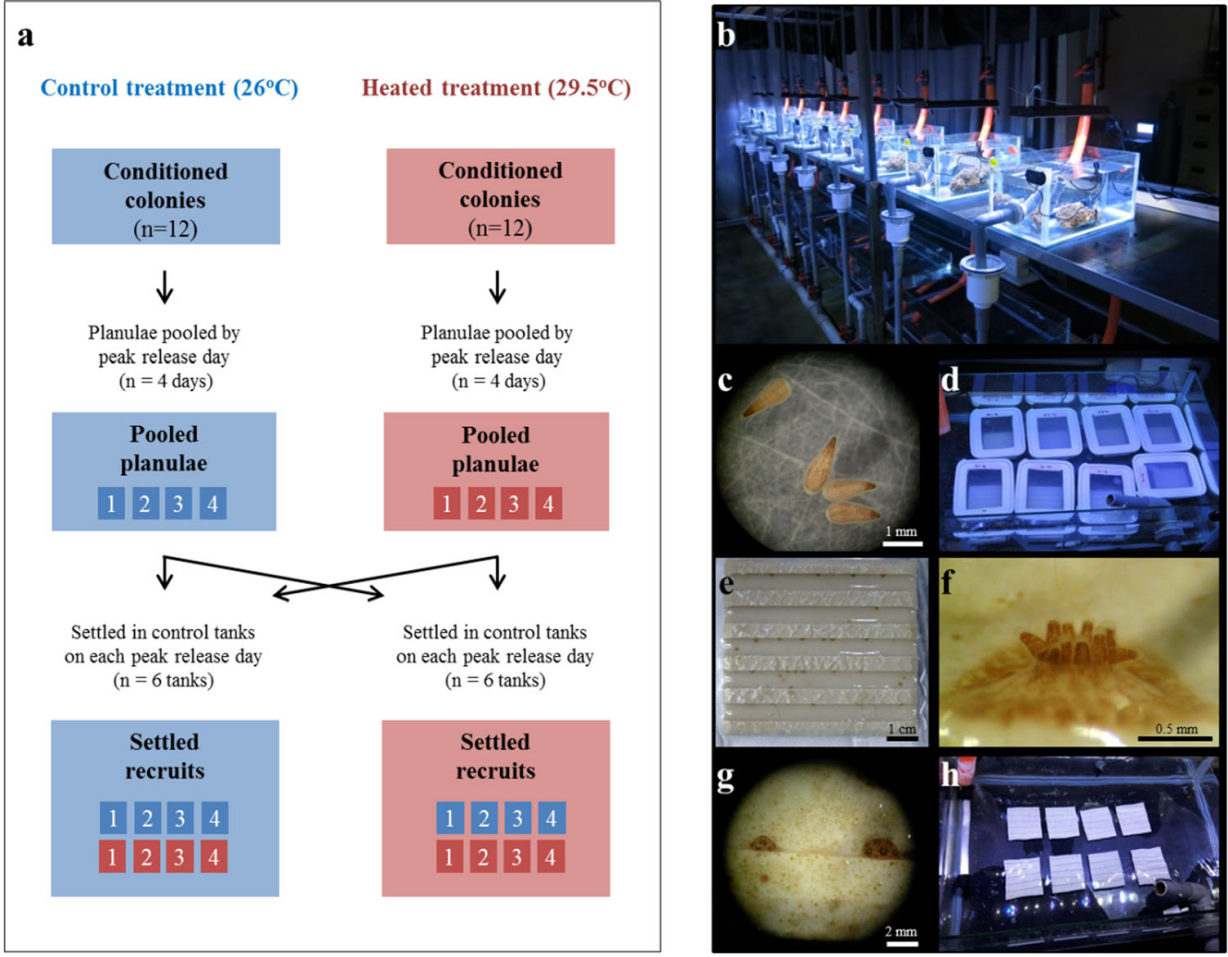
Overview of experimental design. Reproductive timing and planulae production of colonies in control (26 °C) and heated (29.5 °C) treatments were monitored daily across three reproductive cycles (March to May 2017). Planulae size and maximum quantum yield (Fv/Fm) were measured independently across the 4 peak days of reproduction each month. In April and May, planulae were pooled based on parent temperature separately for each of the 4 peak days of reproduction and settled at either 26 °C or 29.5 °C where they grew as recruits. **a** schematic of experimental design; **b** colonies within a flow-through system for the collection of planulae; **c** newly released planulae; **d** recruitment containers used for the first week of planula recruitment; **e** newly settled recruits on a conditioned tile; **f** recruit ~ 1-week post-recruitment; **g** recruits ~ 3-weeks post-recruitment; **h** tiles with recruits held within a recruitment tank

Coral colonies were moved into individual 14-L tanks, in an indoor flow-through experimental system (flow rate: ~ 7 ml s^−1^) (Fig. 1b). Tanks were lit with LED lights (ComboRay G2; Illumagic, Taiwan), with a light intensity of ~ 200 µmol photons m^−2^ s^−1^ (measured using a LI-COR PAR sensor; LICOR Biosciences, USA) on a 12 h:12 h light/dark photoperiod with light exposure from 06:00–18:00, which was similar to natural daylight hours. Sand-filtered seawater ran through an additional 3-tier filtration unit (100, 75, 50 μm) before entering the experimental system. Air temperature was maintained at 26 °C throughout the experiment. Colonies were fed a commercial feed (Coral Frenzy) three times a week.

We randomly assigned colonies to either a control group (26.2 °C ± 0.4; n = 12) or a heated group (29.7 °C ± 0.3; n = 12; Fig. 1a). The control temperature was representative of the spring temperature at Outlet reef (March—May 2017, mean ± SD: 26.7 °C ± 1.2), and the heated temperature was purposely set at a temperature above any experienced in spring but slightly below summer mean temperature at Outlet reef (July—August 2017, mean ± SD: 30.1 ± 1.1) (Fig. S6). Heated tank temperature was controlled in each tank with a 150 W heater; temperature was increased from 26 °C to 29.5 °C (rate: + 0.5 °C / 12 h) over the course of 3 days. We measured colony maximum quantum yield (Fv/Fm), which estimates quantum efficiency of photosystem II, i.e. a measurement of symbiont photochemical performance (Iglesias-Prieto et al. 1992; Jones et al. 2000), every two weeks with a diving PAM (Heinz Walz GmbH, Germany; settings: saturation pulse intensity = 8, measurement light intensity = 8, gain = 2, damp = 2). Prior to Fv/Fm measurements, colonies were dark adapted for 30 min and 3 replicate measurements were taken on each colony. Temperature was recorded every 10 min with HOBO pendant temperature loggers; each colony tank had its own logger (UA-002–08, Onset Computer Corporation, USA) (Fig. S2).

### Reproduction and planulae measurements

The outflow pipe of each colony tank was fitted with a 100 μm-mesh cup to facilitate the collection of coral larvae (hereafter referred to as planulae). We counted the planulae released from each colony daily at ~ 09:00 for three reproductive cycles (March, April and May 2017). Any planula that had settled on the tank or was swimming in the tank (i.e. had not passed into the collection cup) was counted and included in the daily total enumeration for each colony; tanks were scrubbed each day post-counts to ensure accuracy across days. The majority (> 80%) of planulae, however, were collected via the collection cup. We measured planula length and Fv/Fm daily during the four treatment-specific peak days of release for each reproductive cycle; we focussed solely on peak release days because planulae size, respiration rates and susceptibility to stressors are known to differ across the larval release period (Cumbo et al. 2012, 2013). More than two-thirds (71.8% ± 12.9%) of all planulae produced were released each month on these peak days (Table S1). Planula length (*n* = 10 planulae/colony) was measured under a dissecting microscope. We measured only larvae that were elongate and swimming to standardise developmental stage. Fv/Fm was assessed using a diving PAM (settings: saturation pulse intensity = 11, measurement light intensity = 11, gain = 8, damp = 2). Planulae were pooled across colonies based on parent temperature, separately for each of the four peak release days and dark adapted for 30 min. Ten independent Fv/Fm measurements, each containing ~ 15 planulae, were then taken for each temperature treatment group on each of the four days of peak larval release. To do this, we attached a small flexible plastic pipe extension (~ 1 cm in length) to the end of the diving PAM cable, held the cable upright and placed planulae within this pipe extension for each independent measurement. We did not assess whether planulae were the product of sexual or asexual reproduction.

### Recruitment, survival and growth

To examine the recruitment, survival and growth of coral recruits, we again pooled planulae based on parent temperature (treatment pools comprised approximately the same number of planulae from each colony), separately for each of the four peak release days of the April and (separately) May reproductive cycles. For each peak day, pooled planulae from each parent treatment were divided evenly across 12 recruitment containers (with *n* = 30 and *n* = 40 planulae/container for April and May, respectively, across a total of 24 containers). For each parent treatment container set, six of the containers were assigned randomly to one of six 26 °C recruitment tanks (60 L) and the remaining six containers were assigned to one of the six 29.5 °C recruitment tanks. Thus, each recruitment tank held one planula container from each parent temperature–peak release day combination (i.e. 8 containers; see Fig. 1a for experimental design overview). The same recruitment container arrangement was used for both April and May recruitment; each recruitment month used independent recruitment tanks (*n* = 12 recruitment tanks/month).

In each container (volume = 540 cm^3^), we placed one ceramic tile (7 × 7 cm) that had been pre-conditioned for 1 month in a tank rich in crustose coralline algae to facilitate coral recruitment (i.e. planulae attachment to the tile). The plastic containers had meshed (100 μm) sides and lid to facilitate water flow (see Fig. 1d). Recruitment on each tile was recorded daily for one week, after which the tiles were removed from the recruitment containers and placed directly within the recruitment tanks. Recruit survival, growth and Fv/Fm were then monitored 1, 3, 7 and 9 weeks post-recruitment. A recruit was considered dead if it had bleached completely and no polyp was visible under the microscope. Growth was assessed based on changes in recruit diameter, measured under a dissecting microscope (magnification = 10x), over the course of the experiment. Recruit position was mapped on each tile, and each recruit was assigned an individual number to ensure identification over time. Recruits that settled on the edge of the tile were not included in our analyses because it was not possible to measure their size accurately; this was the case for ~ 5% of settled recruits. Fv/Fm of individual recruits was measured using a diving PAM (settings: saturation pulse intensity = 11, measurement light intensity = 11, gain = 8, damp = 2) after careful removal of any surrounding algae; recruits were dark adapted for 30 min prior to measurement. Recruits were fed a commercial feed (Coral Frenzy) three times a week.

### Statistical analyses

We used Rayleigh tests to assess the daily distribution of planulae release for each of the three reproductive cycles. Watson’s tests were used to investigate differences in reproductive timing between treatments each month. We used generalized linear models to assess (1) the effects of parent temperature treatment and colony size on the number of planulae released for each reproductive cycle (with a Poisson distribution) and (2) the effect of parent temperature on planulae Fv/Fm. We used linear mixed-effects models to assess (1) the effect of treatment temperature over time on colony Fv/Fm (with colony as a random effect), (2) the effect of parent treatment temperature on planulae size each month (with colony as a random effect) and (3) the effect of parent temperature and recruitment temperature (and their interaction) over time on recruitment, growth and Fv/Fm of recruits (with tile [*n* = 96/month] nested within recruitment tank [*n* = 12 tanks/month] as random effects; recruitment analyses only used tank as a random effect). Recruit survival was assessed using Cox mixed-effects models with tile nested within recruitment tank as random effects. All analyses were done in R (R Core Team 2019) using the packages circular (Agostinelli and Lund 2017), car (Fox and Weisberg 2019), lme4 (Bates et al. 2015), lmerTest (Kuznetsova et al. 2017), MuMIn (Barton 2009), survival (Therneau 2015), survminer (Kassambara et al. 2019) and coxme (Therneau 2020).

## Results

### Colony reproductive timing and planula abundance

**Fig. 2:**
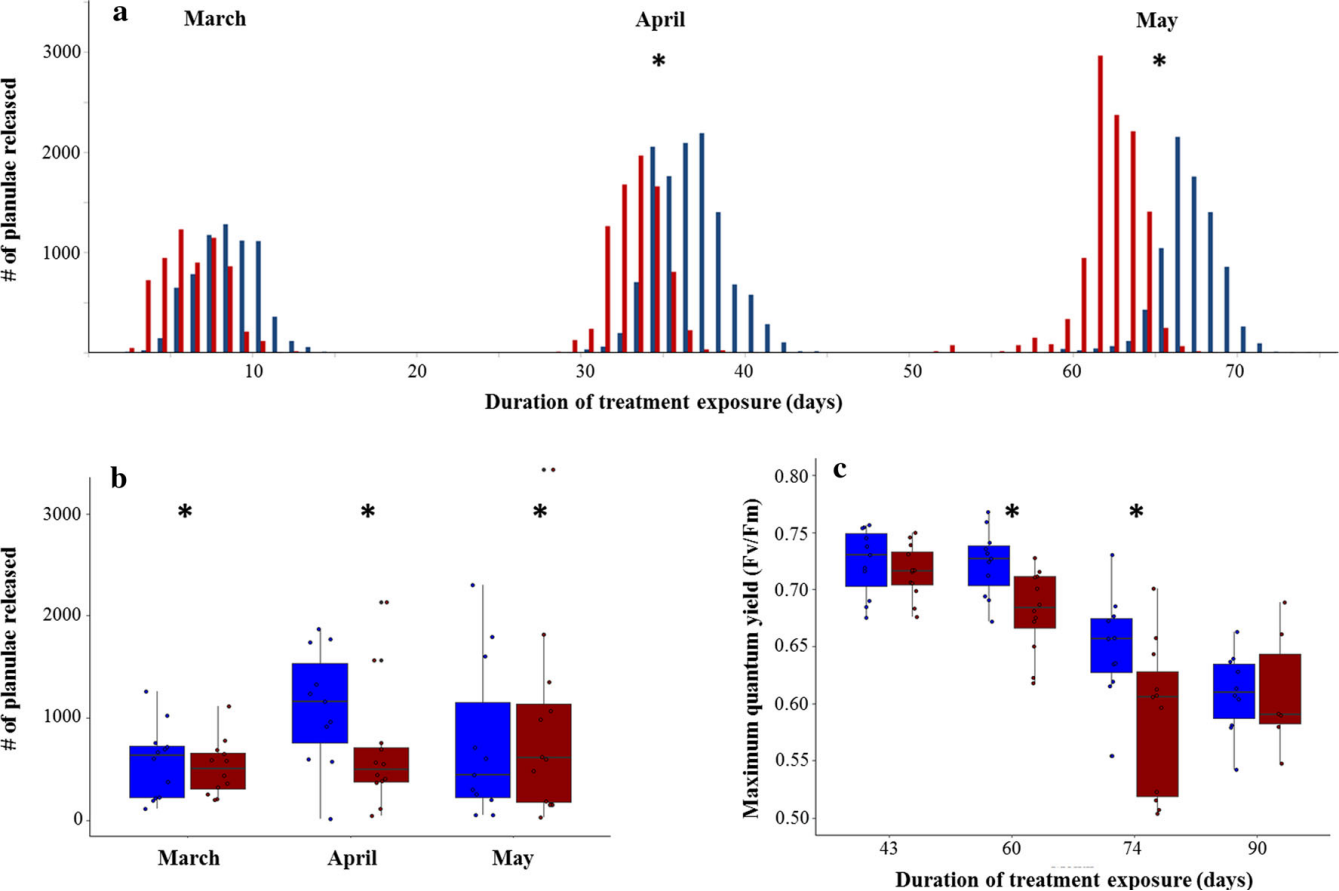
Response of adult *Pocillopora acuta* coral colonies held within control (26 °C; blue) and heated (29.5 °C; red) treatments across three reproductive cycles (March to May 2017). **a** Total number of planulae released in each month across all colonies in each treatment for each day of the experiment; **b** mean number of planulae released per colony each month; **c** colony maximum quantum yield (Fv/Fm). No data are available for March due to equipment malfunction. The line in the boxplots shows the median value, and the bottom and top of the box represent the 25th and 75th quartile ranges, respectively. Asterisks indicate significant differences between parental temperature treatments

The cumulative duration of exposure of coral parent colonies to temperature treatments at the onset of each reproductive cycle was approximately 5 days (Month 1; March), 30 days (Month 2: April) and 60 days (Month 3: May). There was a unimodal distribution of planula release within both temperature treatments for all months (Rayleigh tests, *p* < 0.001) (Table 1; Fig. S3). There was no difference in reproductive timing between temperature treatments in March; however, in April and May, colonies held at 29.5 °C released planulae significantly earlier in the lunar cycle (Watson’s tests, April: F = 0.63, p < 0.001; May: F = 0.94, *p* < 0.001) (Fig. 2a, Table 1). For colonies held at 26 °C (control), there was no difference in reproductive timing between March and May, but planulae were released earlier in the lunar cycle in April (compared to March and May) (Table S2). For colonies held at 29.5 °C, planulae were released earlier in the lunar cycle in April and May (compared to March), but there was no difference in timing between April and May (Table S2).

**Table 1:** Timing of planula release by *Pocillopora acuta* from March to May 2017, converted to mean angle in circular distributions (see Fig. S3) and results of Rayleigh tests for uniformity of distribution and Watson’s tests for homogeneity of reproductive timing by coral colonies held at either 26 °C or 29.5 °C. Sample size is 11 for the control treatment in April and May due to the death of one colony. Lunar day 1 refers to the new moon

Treatment	N	Mean angle	Mean lunar day	Rayleigh test r	*P*-value	Watson’s test F	*P*-value
March							
26 °C 29.5 °C	12 12	116 89	9.34 7.17	0.84 0.87	< 0.001 < 0.001	0.18	> 0.05
April							
26 °C 29.5 °C	11 12	90 48	7.25 3.87	0.74 0.88	< 0.001 < 0.001	0.63	< 0.001
May							
26 °C 29.5 °C	11 12	98 44	7.89 3.54	0.82 0.82	< 0.001 < 0.001	0.94	< 0.001

Colonies in the heated treatment released fewer planulae than control colonies in March and April (mean ± SE, March: control 571 ± 103, heated: 516 ± 79, April: control 1160 ± 173, heated: 671 ± 173; generalized linear model, March: z = − 6.59, *p* < 0.001; April: z = − 36.85, *p* < 0.001), but produced more in May (mean ± SE, May: control 693 ± 223, heated: 908 ± 278; generalized linear model, z = 12.24, *p* < 0.001) (Fig. 2b; Table S3). Greater reproduction in May appears to be due to one colony in the heated treatment that produced ~ 3400 planulae, much higher than the average for other colonies in the same treatment. When this outlier colony was removed from the May analysis, colonies in the control treatment produced more planulae (mean ± SE, control 756 ± 225, heated: 678 ± 172; generalized linear model, z = − 6.90, *p* < 0.001; Table S3). Colony size had a significant effect on the number of planulae released across all months, whereby larger colonies produced more planulae (generalized linear models, March: z = 21.13, *p* < 0.001; April: z = 30.75, *p* < 0.001; May: z = 6.26, *p* < 0.001; May with outlier removed: z = 2.30, *p* = 0.003) (Fig. S4; Table S3). There was no difference in colony diameter between treatments (mean ± SE; 26 °C: 13.50 cm ± 0.59 cm, 29.5 °C: 13.62 cm ± 0.65 cm; Mann–Whitney test, W = 82.5, *p* = 0.56). Colonies in the heated treatment had lower Fv/Fm (linear mixed-effects model, t = − 3.07, *p* = 0.006) and Fv/Fm decreased over time in both treatments (linear mixed-effects model, t = − 9.37, *p* < 0.001) (Fig. 2c; Table S4).

### Planula size, Fv/Fm and recruitment

**Fig. 3:**
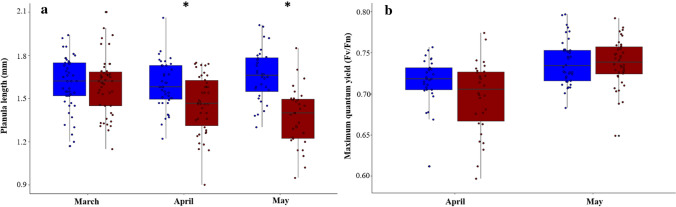
Response of *Pocillopora acuta* planulae released from colonies held within control (26 °C; blue) and heated (29.5 °C; red) treatments across three reproductive cycles (March to May 2017). **a** Mean length of planulae. Asterisks indicate significant differences between parental temperature treatments; **b** Mean maximum quantum yield of planulae (Fv/Fm); no data are available for March due to equipment malfunction. The line in the boxplots shows the median value, and the bottom and top of the box represent the 25th and 75th quartile ranges, respectively

In March, there was no difference in the size of planulae released by adult corals held at either temperature (linear mixed-effects model *p* > 0.05; Table S5). However, in both April and May, planulae released by adults held at 29.5 °C were significantly smaller than those released from colonies at 26 °C (linear mixed-effects models, April: t = − 2.77, *p* = 0.013; May: t = − 5.71, *p* < 0.001) (Fig. 3a; Table S5). The incorporation of colony as a random effect in the planulae size models increased the R^2^ value by 0.16 (March), 0.12 (April) and 0.11(May), indicating a parental colony effect. There was no difference in Fv/Fm between planulae released by adults under different temperature treatments (generalized linear models, April: *p* > 0.05; May: *p* > 0.05; Fig. 3b; Table S6). After 6 days post-release, the proportion of planulae that settled did not differ based on parent treatment temperature (for either recruitment month), but was lower in the heated recruitment tanks in April (linear mixed-effects models, t = − 2.35, *p* = 0.026) (Fig. S5, Table S7).

### Recruit survival, size and Fv/Fm

**Fig. 4:**
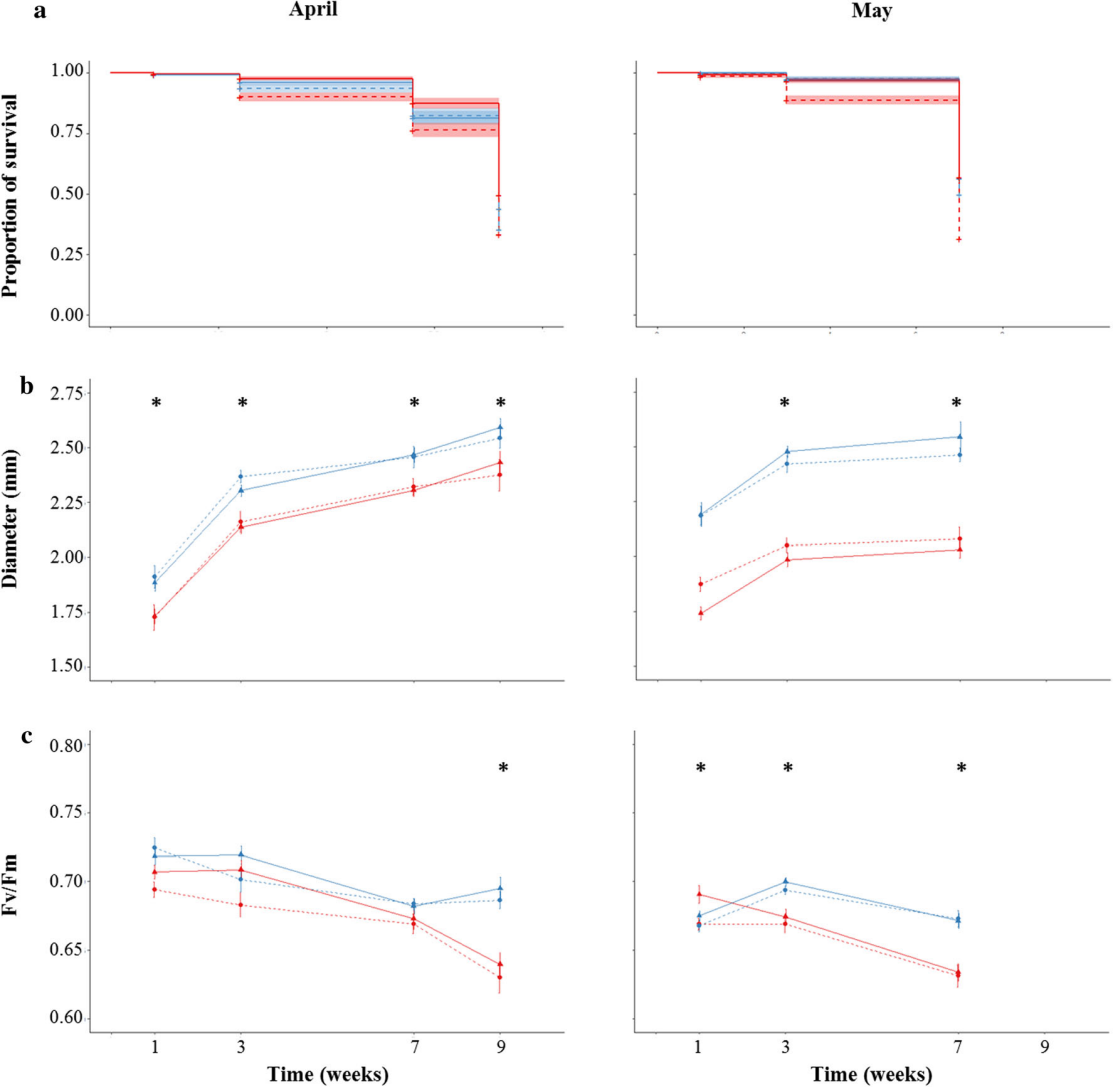
**a** Survival, **b** size and **c** Fv/Fm of *Pocillopora acuta* coral recruits produced by parent colonies held in control (26 °C; blue lines) or heated (29.5 °C; red lines) water and then grown in control (26 °C; solid lines) and heated (29.5 °C; dashed lines) recruitment tanks. Recruits sourced from the April and May planulation periods were held in independent recruitment tanks (*n* = 12 tanks/month). Data for week 9 in May were not included due to small sample size. Asterisks indicate significant parent treatment effect; there were no significant recruitment temperature effects (see Tables S9, S10, S12-15)

Recruit survival did not differ based on parent or recruitment temperature in April. In May, however, survival of recruits sourced from adults held at 29.5 °C was lower when they settled at 29.5 °C than when they settled at the control temperature (Cox mixed-effects model, z = 2.86, *p* = 0.004) (Fig. 4a; Table S8). No difference in survival based on recruitment temperature was observed for recruits sourced from adults held at 26 °C. Recruits sourced from parent colonies in the 29.5 °C treatment were significantly smaller (linear mixed-effects models, April: t = − 5.08, *p* < 0.001; May: t = − 14.14, *p* < 0.001) (Fig. 4b; Table S9) and had lower Fv/Fm (linear mixed-effects models, April: t = − 4.23, *p* < 0.001; May: t = − 3.65, *p* = 0.002) (Fig. 4c; Table S10) than recruits sourced from adults in the control treatment. Recruitment temperature did not affect recruit size or Fv/Fm in either recruitment month (linear mixed-effects models, *p* > 0.05) (Tables S9 & S10). The interaction between parent and recruitment temperature was significant in May for recruit size (linear mixed-effects model, t = 2.83, *p* < 0.001), with recruits sourced from heated adults being larger when grown at 29.5 °C, than at 26 °C, while the reverse was observed for recruits sourced from adults held at ambient temperature (Fig. 4b). In both months, recruit size increased over time (April: t = 35.22, *p* < 0.001; May: t = 16.01, *p* = 0.006) and Fv/Fm decreased (April: t = − 19.97, *p* < 0.001; May: t = − 9.98, *p* < 0.001) (Tables S9 & S10).

## Discussion

To evaluate the potential capacity for transgenerational acclimation in corals, we examined the physiological and demographic responses to temperature of adult *P. acuta* colonies, planulae and recruits. Thermal conditioning at 29.5 °C led to earlier reproduction, reduced reproductive investment and lower symbiont photochemical performance associated with adult colonies. Planulae released from thermally conditioned adults were smaller than those from control (26 °C) parents, but Fv/Fm was similar. Recruitment did not differ based on parent treatment but was lower at the heated recruitment temperature in the second month of the experiment. Recruit survival was not significantly affected by either parent or recruitment temperature. However, recruits sourced from thermally conditioned parents were smaller and had lower Fv/Fm by the end of the experiment. Taken together, our results do not support the notion that thermal conditioning of *P. acuta* adult colonies sourced from a chronically warmed reef benefits offspring when they settle and grow in warm conditions.

### Reproductive timing, planulae investment and recruitment

Coral colonies held in warmer water reproduced earlier. On average, larval release by heated colonies was 3.4 days (April) and 4.4 days (May) earlier than by control colonies. Shifts to earlier reproduction in response to warming have been observed in brooding and spawning corals, both experimentally (Crowder et al. 2014; Paxton et al. 2016) and in the wild (Nozawa 2007; Fan et al. 2017). Typically, reproductive timing is controlled by specific environmental cues (e.g. lunar irradiance, daylight cycles, sea surface temperature, wind fields), which can vary across species and regions (Fan et al. 2006; Brady et al. 2009; van Woesik 2010; Harrison et al. 2011; Lin and Nozawa 2017). In some cases, changes in reproductive timing can be a response to an acute stress event (e.g. high nutrient conditions; Cox and Ward 2002; temperature perturbations from upwelling; Tew et al. 2014). Climate change-induced warming, however, is chronic stressor, and a more permanent shift to earlier reproduction could lead to incomplete (or faster) gametogenesis and/or a mismatch between the timing of planula release and of optimal conditions for larval survival (Shlesinger and Loya 2019).

Thermally conditioned colonies produced fewer offspring than colonies held at 26 °C. Decreased fecundity under thermal stress has been found across a wide range of coral species (McClanahan et al. 2009; Paxton et al. 2016; Howells et al. 2016a, b) and is underpinned at a physiological level by increased metabolism and depleted adult lipid reserves (Grottoli et al. 2004; Rodrigues et al. 2008). Indeed, symbiont photochemical performance (Fv/Fm) decreased over the course of the experiment and significantly more so in colonies held in the heated treatment. Similar decreases in colony Fv/Fm in other transgenerational acclimation experiments, both between treatments (Putnam and Gates 2015) and over time (Bellworthy et al. 2019), underscore the limitations of thermal conditioning of adult colonies: long-term reproduction in a laboratory setting is finite due to declines in adult health (but see Craggs et al. 2020).

Planulae released by thermally conditioned adults were smaller than those produced by corals under control temperatures. This smaller size (see also Putnam and Gates 2015), coupled with the reduced fecundity of heated adults, points to significantly reduced reproductive investment by corals exposed to high temperature. It is not clear whether small size would be an advantage or a detriment to coral larvae. On one hand, the high size-specific metabolic rate of small coral planulae brooded by parents exposed to high temperature could hasten recruitment and post-recruitment growth (Putnam and Gates 2015). On the other hand, smaller larvae can contain a smaller amount of lipids than larger larvae (e.g. de Putron et al. 2017) and the rapid use of these limited lipid reserves at increased temperatures (Rivest and Hofmann 2015) could limit the time window for pelagic dispersal, larval ability to find suitable habitat and ultimately recruitment success (Richmond 1987; Isomura and Nishihira 2001). Moreover, small larvae may turn into small recruits and recruit survival has been related to size (e.g. Raymundo and Maypa 2004). There was no difference in Fv/Fm in planulae sourced from control or heated parents (see also Putnam et al. 2008; Bellworthy et al. 2019 for similar Fv/Fm of planulae at different temperatures). The similarity of planula Fv/Fm in our experiment suggests that the photosynthetic capacity between treatments is likely equivalent. However, it is important to note that size does not necessarily have a significant effect on the density of Symbiodiniaceae within planulae (Isomura and Nishihira 2001; *P. damicornis*) and that Symbiodiniaceae do not play a large role in the provision of energy to pelagic planulae (Kopp et al. 2016).

Final recruitment (i.e. the total number of recruits settled on a tile after 6 days) was lower at higher temperature, regardless of parental temperature in the April recruitment and unaffected by temperature in the May recruitment. Increased temperature can have a variable influence on recruitment, with evidence of no effect of parental (Bellworthy et al. 2019) or recruitment temperature (Anlauf et al. 2011; Chua et al. 2013), or increased mortality following initially high recruitment (Nozawa and Harrison 2007). The lack of a consistent recruitment disadvantage across the two recruitment months and the absence of a parent temperature effect suggest that the treatment temperatures used in this study were within an acceptable range for recruitment for corals sourced from Outlet reef (see Fig. S6).

### Offspring response to parental thermal conditioning: recruit survival, growth and symbiont photochemical efficiency

Neither parent nor recruitment temperature affected recruit survival, but recruit size and symbiont photochemical performance were affected by parental temperature. Mortality of recruits in both temperature treatments generally increased over time, especially in the May recruitment, but this is typical of this early recruit life stage (Sato 1985). In May, survival of recruits sourced from heated adults was lower at the heated temperature than at the control recruitment temperature—a pattern not observed for recruits sourced from adults held in control conditions and also not expected if transgenerational acclimation confers resistance to offspring exposed to elevated temperatures. Recruits from adults held at 29.5 °C were smaller and had a lower Fv/Fm compared to their counterparts sourced from control parents. These recruits, which were on average smaller as planulae, were therefore unable to ‘catch up’ to the size of the control-sourced recruits regardless of recruitment temperature, perhaps because of an overall lower energy provision from Symbiodiniaceae (i.e. as suggested by their lower Fv/Fm). Being a small recruit on a reef can be detrimental, as risk of predation and algal overgrowth is greater for smaller corals and can ultimately lead to higher mortality (Edmunds et al. 2004; Raymundo and Maypa 2004; Vermeij 2006). Our results highlight an influential, detrimental effect of adult temperature on offspring rather than the beneficial effect expected from transgenerational acclimation. Furthermore, our experiment demonstrates the need to assess recruit performance over weeks, not days, to elucidate clear parent temperature effects.

### Could the lack of transgenerational acclimation be due to the thermal condition of the source reef?

Chronic warming influences coral thermal tolerance (Howells et al. 2012; Fine et al. 2013; Silbiger et al. 2019). Owing in part to the warm effluent of the nearby nuclear plant and in part to the unique oceanographic characteristics (i.e. high frequency and magnitude upwelling) of southern Taiwan, Outlet Reef has experienced warmer than average temperatures and higher summer extremes than surrounding reefs for over three decades. There is evidence that this chronic thermal exposure has affected corals in the area. Shifts to more resistant Symbiodiniaceae have been observed across multiple coral species (Keshavmurthy et al. 2012, 2014), and thermal acclimation of *P. damicornis* from Nanwan Bay has been noted (Mayfield et al. 2013). The result might have been selection for corals that survive better at relatively high temperatures. If this were so, our high temperature treatment might not have been stressful enough to coral colonies to elicit a thermal conditioning effect. Moreover, thermal adaptation can limit plasticity, such that the capacity for cross-generational acclimation might have been eroded. Surprisingly, areas characterized by elevated and variable temperatures (i.e. sites thought to harbour species with high acclimation potential) may actually be most at risk because their capacity to acclimate beyond their already high upper thermal limits has been exceeded (Tomanek 2008). Evidence for reduced thermal plasticity at warm and variable intertidal/subtidal sites has been found across several marine molluscs and crustaceans (Stenseng et al. 2005; Gilman et al. 2006; Somero 2010) and recently in corals from variable environments (Klepac and Barshis 2020). It is therefore possible that our parent colonies, from a chronically warm and thermally variable reef, lacked the capacity for further thermal plasticity.

That being said, our experimental colonies did respond to chronic warming. We exposed them to high temperature at a time of year when they would be unlikely to experience such extremes (Fig. S6), and we did find a shift in timing of reproduction, lower planula size and number, and clear effects of parent temperature on recruit size and Fv/Fm. These results suggest that our high temperature treatment did have some capacity to affect adult, larvae and recruits, even in a population potentially selected for thermal tolerance.

## Conclusions

We found that the thermal environment of parent colonies has implications for adult corals as well as planulae and recruits. Thermal conditioning of adults led to smaller planulae, smaller recruit size and reduced symbiont photochemical performance associated with recruits. There was no evidence of improved offspring performance (i.e. higher survival) that would suggest enhanced resistance as a result of transgenerational acclimation. We did not assess other components of the coral holobiont (e.g. influence of *Symbiodinouim*, bacteria, or viruses; see Torda et al. 2017) that could affect planula and recruit growth and survival. We also examined a single generational effect of adult conditioning, which might be insufficient to comprehensively document the capacity for epigenetic effects over the long term (Donelson et al. 2018). Nevertheless, for transgenerational acclimation to offer hope for coral persistence, its effects need to be large, positive and relatively rapid. Our results suggest that this is not the case, at least for the species and conditions we examined.

By focusing mainly on demographic responses of the filial generation, we have provided a simple and relatively cost-effective method to probe for transgenerational acclimation potential without the need to commit to highly technical physiological and molecular techniques. It is likely that the responses of corals to thermal conditioning are variable across species and regions and it may be fruitful to explore this diversity in situations where the ecological costs of collection and experimentation do not outweigh the benefits (i.e. with a small sample size of colonies sourced from healthy natural populations, aquarium-based populations, or within existing restoration projects). The idea of using transgenerational acclimation to ‘save coral reefs’ is polarizing (Braverman 2016; Abelson 2020). However, the slow progress made in tackling the root cause of coral loss, i.e. climate change (Hoegh-Guldberg et al. 2017; Hughes et al. 2017b) and the inevitable committed warming of the ocean, even under optimistic emissions scenarios, seem to justify a diversity of efforts to explore how to enhance coral resilience.

## Supplementary Information

Below is the link to the electronic supplementary material.

Supplementary file1 (DOCX 725 kb)
