# Supplementary material for: Effects of thermal conditioning on the performance of Pocillopora acuta adult coral colonies and their offspring
Source: Coral Reefs. 2021 Jul 21;40(5):1491–503. doi: 10.1007/s00338-021-02123-9 (PMC8550305; doi:10.1007/s00338-021-02123-9)
Supplement: Supplementary file 1 — Supplementary file1 (DOCX 725 kb) [file 338_2021_2123_MOESM1_ESM.docx]

**Supplementary materials**


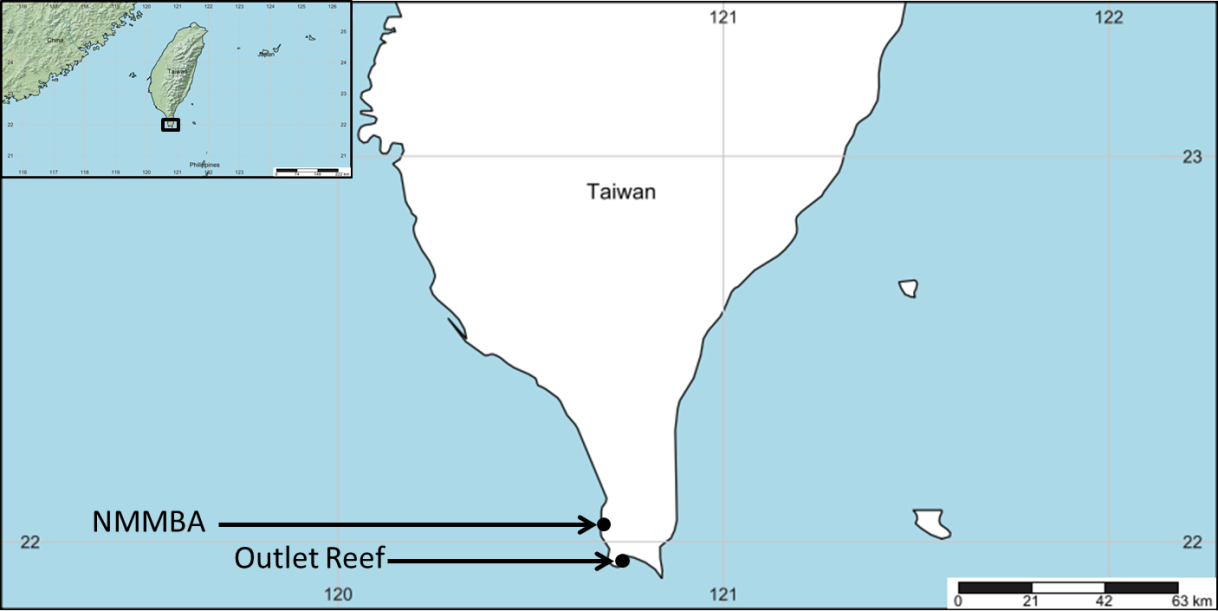


**Figure S1**: Colony collection site (Outlet Reef) and research center site (National Museum of Marine Biology & Aquarium; NMMBA) in southern Taiwan.


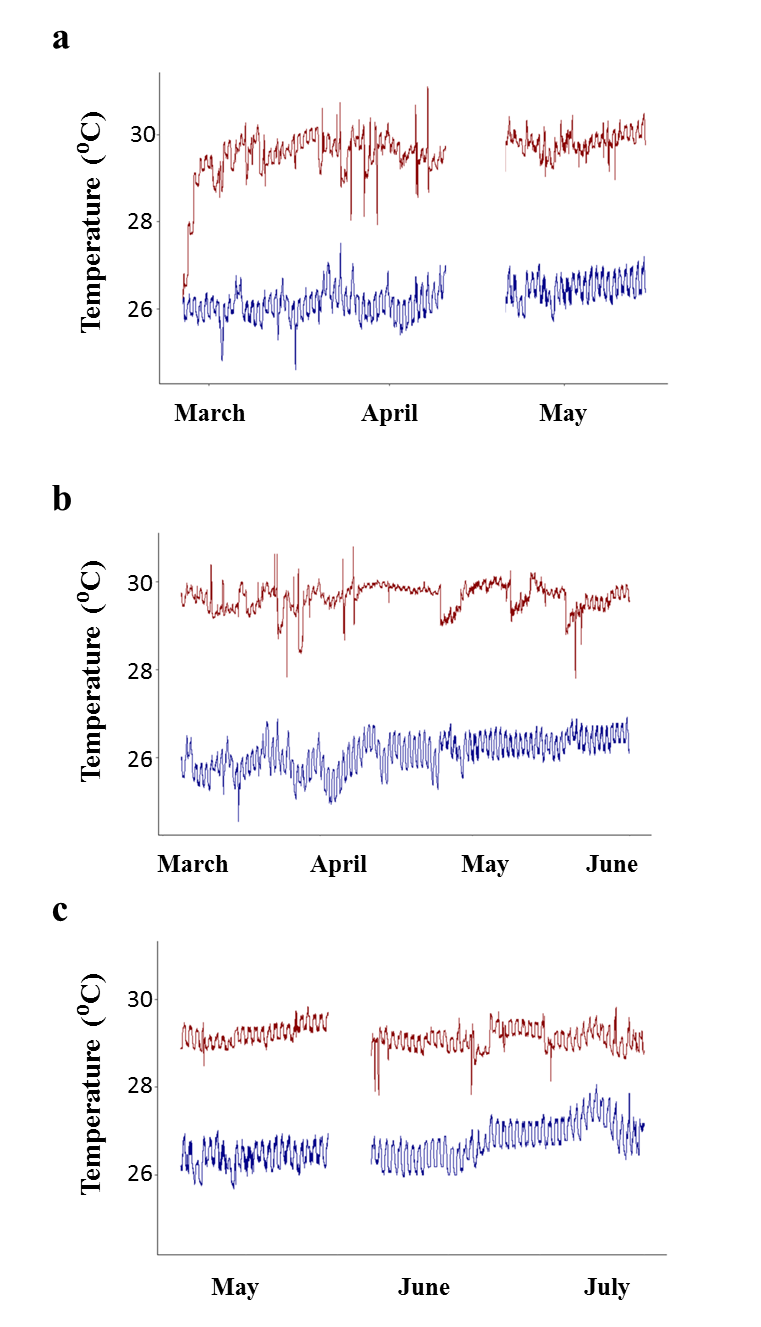


**Figure S2**: Mean temperature in the control (blue lines) and heated (red lines) treatments for the (a) parent colony tanks (heated: 29.7°C ± 0.3; control: 26.3°C ± 0.4), (b) April recruit recruitment tanks (heated: 29.7°C ± 0.3; control: 26.1°C ± 0.4), and (c) May recruit recruitment tanks (heated: 29.2°C ± 0.2; control: 26.7°C ± 0.4). No temperature data are available from 10-20 April for the parent colony tanks, and 13-19 May in the May recruit tanks due technical issues.


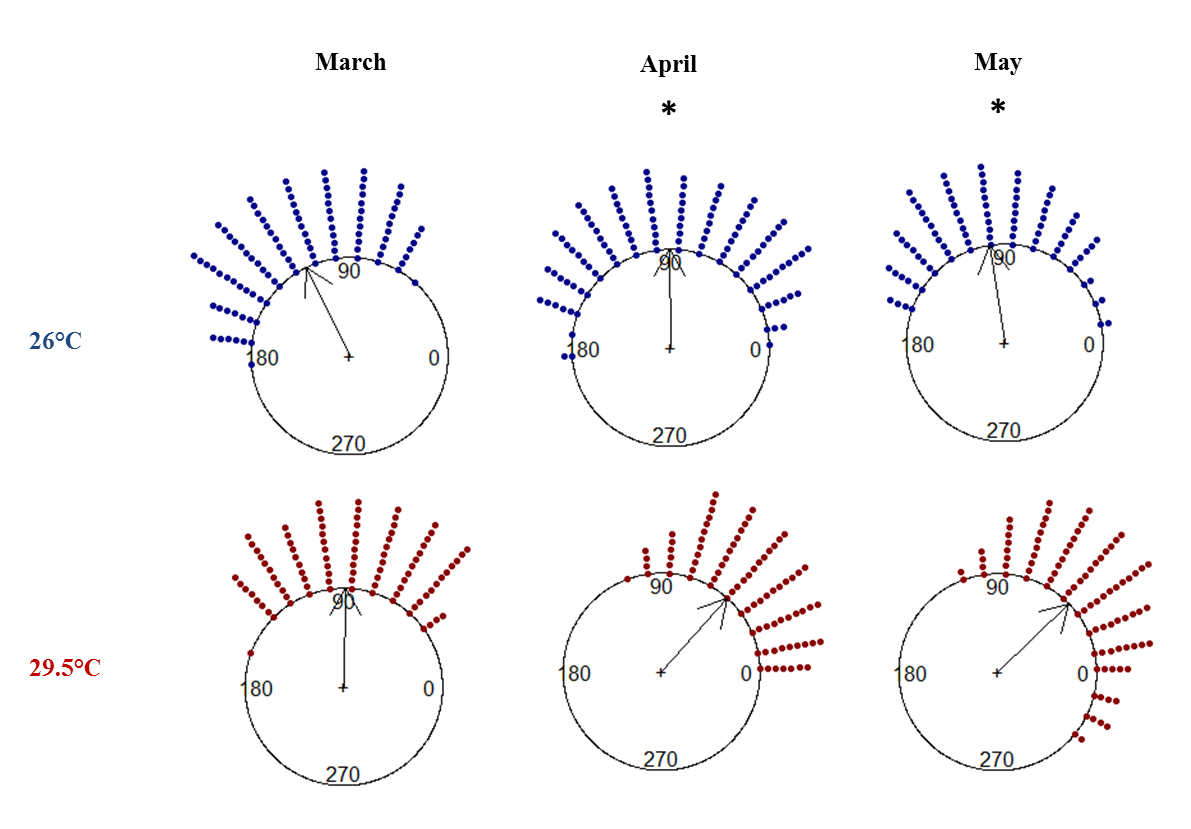


**Figure S3.** Circular plots of reproductive timing of *Pocillopora acuta* across three reproductive cycles when held at either 26°C or 29.5°C from March to May 2017. Approximate cumulative duration of treatment exposure was 5 days (March), 30 days (April), and 60 days (May). Asterisks indicate significant differences between treatments (Watson’s tests, p<0.001).

**
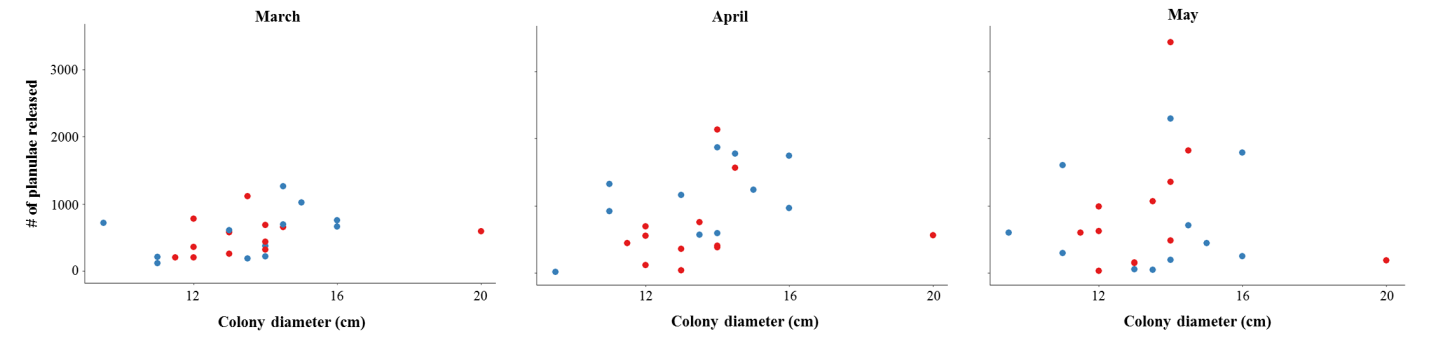
**

**Figure S4**: Relationship between total number of planulae released and colony diameter for adult *Pocillopora acuta* corals exposed to control (26°C; blue dots) and heated (29.5°C; red dots) treatments from March to May 2017.

**
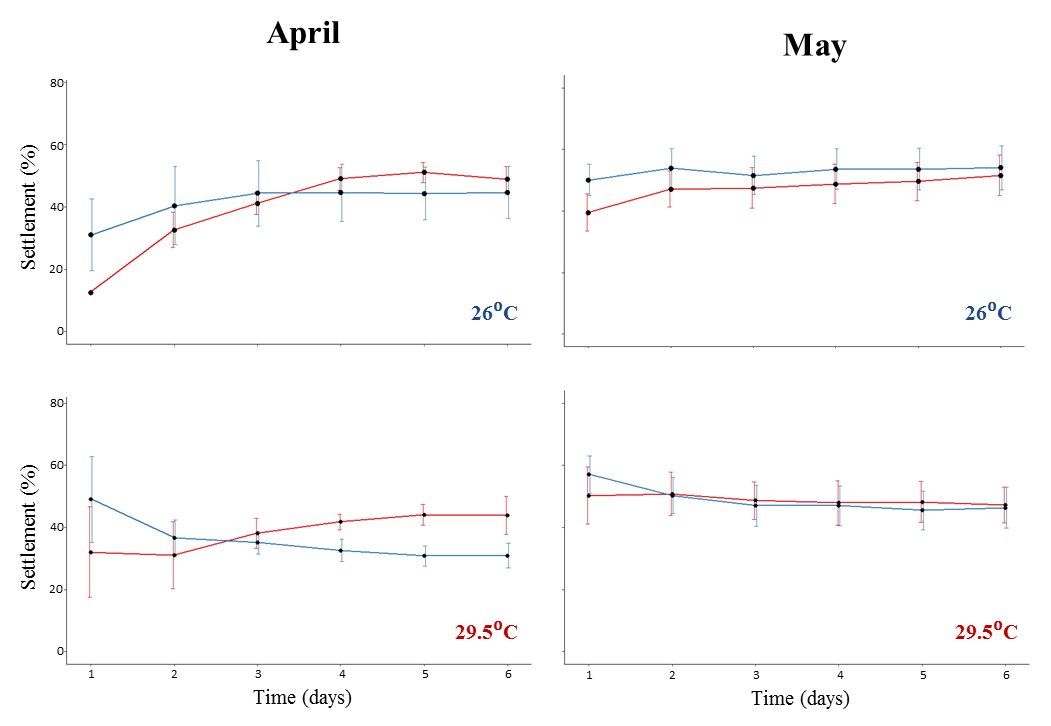
**

**Figure S5.** Percentage of *Pocillopora acuta* planulae (mean ± SE) released that settled within control (26°C) and heated (29.5°C) recruitment tanks within the first 6 days post-release. Planulae were sourced from colonies held within the control treatment (blue lines) and heated treatment (red lines); independent recruitment was conducted for the April and May planulation.

**
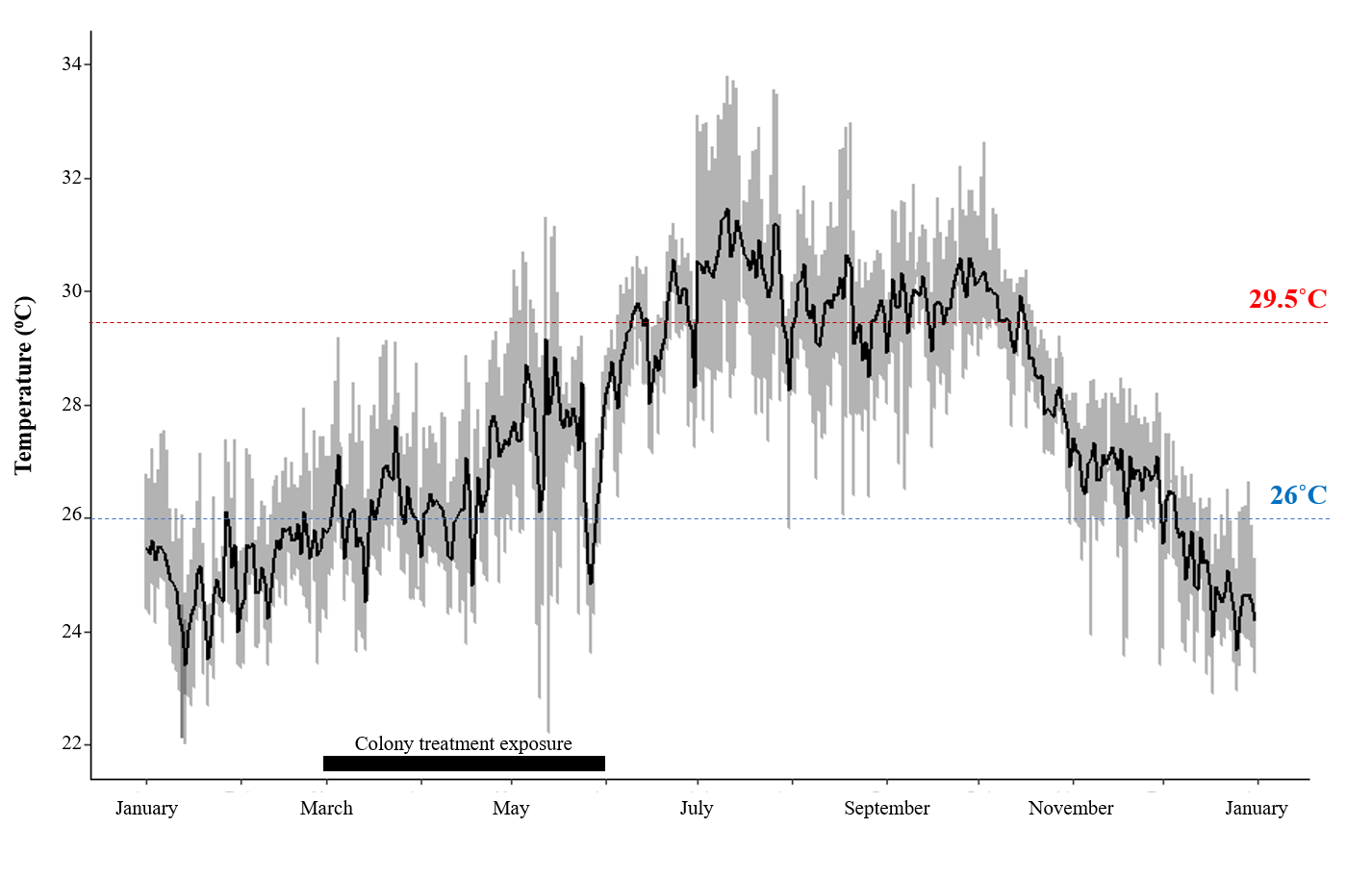
**

**Figure S6.** Temperature at Outlet reef from January 2017- January2018; temperature recorded every 10 minutes (grey line), experimental treatment temperatures (blue: control; red: heated) and daily mean temperature (black line).

**Table S1**. Number of days of planula release by *Pocillopora acuta* from March to May 2017 within treatments (control: 26°C; heated: 29.5°C), and proportion of release occurring in the 4 peak days each month. Lunar day 1 refers to the new moon.

| **Treatment** | **Number of reproductive days each month** | **Proportion of reproduction within the 4 peak days** |
| --- | --- | --- |
| **March** |  |  |
| 26°C  29.5°C | 8.75 ± 1.7  7.92 ± 1.3 | 0.71 ± 0.14  0.65 ± 0.07 |
| **April** |  |  |
| 26°C  29.5°C | 10.45 ± 3.6  6.92 ± 2.1 | 0.51 ± 0.24  0.85 ± 0.10 |
| **May** |  |  |
| 26°C  29.5°C | 8.09 ± 3.0  7.75 ± 3.1 | 0.75 ± 0.13  0.84 ± 0.11 |

**Table S2**. Results of Watson’s tests for homogeneity of *Pocillopora acuta* reproductive timing within parent treatments across three reproductive cycles (March to May 2017).

| **Treatment** | **N** | **F** | **P** |
| --- | --- | --- | --- |
| **Control** (26°C) |  |  |  |
| March vs. April  April vs. May  March vs. May | 12, 11  11, 11  12, 11 | 0.30  0.23  0.06 | **<0.01**  **<0.05**  >0.10 |
| **Heated** (29.5°C) |  |  |  |
| March vs. April  April vs. May  March vs. May | 12, 12  12, 12  12, 12 | 0.52  0.12  0.58 | **<0.001**  >0.10  **<0.001** |

**Table S3**. Effect of coral colony size and experimental temperature on the number of *Pocillopora acuta* planulae released per month from March to May 2017. Parameters were obtained from a generalized linear model (with Poisson distribution). Experimental temperatures were 26°C (control) and 29.5°C (heated).

| **Fixed effects** | **Estimate** | **Standard**  **error** | **z** | **Lower**  **CI** | **Upper**  **CI** | **P** |
| --- | --- | --- | --- | --- | --- | --- |
| **March** |  |  |  |  |  |  |
| Intercept  Treatment  Colony size | 5.204  -0.116  0.084 | 0.056  0.018  0.004 | 92.866  -6.586  21.134 | 5.094  -0.150  0.076 | 5.314  -0.081  0.092 | <0.001  **<0.001**  **<0.001** |
| **April** |  |  |  |  |  |  |
| Intercept  Treatment  Colony size | 5.685  -0.531  0.097 | 0.045  0.014  0.003 | 127.244  -36.852  30.748 | 5.597  -0.559  0.091 | 5.772  -0.503  0.104 | <0.001  **<0.001**  **<0.001** |
| **May** |  |  |  |  |  |  |
| Intercept  Treatment  Colony size | 6.345  0.178  0.021 | 0.047  0.015  0.003 | 135.54  12.237  6.258 | 6.253  0.150  0.014 | 6.436  0.207  0.028 | <0.001  **<0.001**  **<0.001** |
| **May** (outlier removed) |  |  |  |  |  |  |
| Intercept  Treatment  Colony size | 6.483  -0.109  0.011 | 0.052  0.016  0.004 | 126.87  -6.890  2.298 | 6.382  -0.141  0.004 | 6.583  -0.079  0.018 | <0.001  **<0.001**  **0.003** |

**Model**: Reproductive abundance ~ treatment + colony size

**Table S4**. The effect of experimental temperatures (26°C; control and 29.5°C; heated) on colony Fv/Fm over time. Parameters were obtained from a linear mixed effect model, with colony as a random effect. Measurements were taken at 43, 60, 74, and 90 days of treatment exposure; no earlier time points were included due to equipment malfunction.

| **Fixed effects** | **Estimate** | **Standard**  **error** | **t** | **Lower**  **CI** | **Upper**  **CI** | **P** |
| --- | --- | --- | --- | --- | --- | --- |
| Intercept  Treatment  Time | 0.676  -0.031  -0.048 | 0.007  0.010  0.005 | 97.647  -3.067  -9.367 | 0.663  -0.050  -0.058 | 0.690  -0.011  -0.038 | <0.001  **0.006**  **<0.001** |

**Model**: Colony Fv/Fm ~ treatment + scale (time) + (1| colony)

**Table S5**. Linear mixed effect model output for *Pocillopora acuta* planulae length for March to May 2017; fixed effect was parent temperature, and random effect was parent colony (n = 12 colonies/treatment). The number of individual planulae measured each month were: March: n = 893, April: n = 761, and May: n = 734.

| **Fixed effects** | **Estimate** | **Standard**  **error** | **t** | **Lower**  **CI** | **Upper**  **CI** | **P** |
| --- | --- | --- | --- | --- | --- | --- |
| **March** |  |  |  |  |  |  |
| Intercept  Parent temp. | 1.601  -0.002 | 0.042  0.060 | 37.822  -0.032 | 1.518  -0.119 | 1.684  0.115 | <0.001  0.975 |
| **April** |  |  |  |  |  |  |
| Intercept  Parent temp. | 1.600  -0.140 | 0.037  0.051 | 43.435  -2.765 | 1.525  -0.239 | 1.670  -0.041 | <0.001  **0.013** |
| **May** |  |  |  |  |  |  |
| Intercept  Parent temp. | 1.682  -0.308 | 0.038  0.054 | 44.365  -5.711 | 1.608  -0.414 | 1.757  -0.202 | <0.001  **<0.001** |

**Model**: Planulae length ~ parent treatment + (1| colony)

**Table S6**. Generalized linear model output for *Pocillopora acuta* planulae Fv/Fm for April and May 2017 (no data available for March due to equipment malfunction).

| **Fixed effects** | **Estimate** | **Standard**  **error** | **t** | **Lower**  **CI** | **Upper**  **CI** | **P** |
| --- | --- | --- | --- | --- | --- | --- |
| **April** |  |  |  |  |  |  |
| Intercept  Parent temp. | 0.715  -0.019 | 0.007  0.010 | 105.507  -1.944 | 0.702  -0.374 | 0.729  0.0002 | <0.001  0.057 |
| **May** |  |  |  |  |  |  |
| Intercept  Parent temp. | 0.738  -0.001 | 0.004  0.006 | 166.153  -0.091 | 0.730  -0.013 | 0.747  0.012 | <0.001  0.927 |

**Model**: Planulae Fv/Fm ~ parent treatment

**Table S7**. Linear mixed effect model output for the proportion of *Pocillopora acuta* planulae that settled after 6 days in April and May 2017. Fixed effects were parent temperature, recruitment temperature (and parent: recruitment temperature interaction). A random effect of tank was included; 12 tanks were used each month.

| **Fixed effects** | **Estimate** | **Standard**  **error** | **t** | **Lower**  **CI** | **Upper**  **CI** | **P** |
| --- | --- | --- | --- | --- | --- | --- |
| **April** |  |  |  |  |  |  |
| Intercept  Parent temp.  Recruitment temp.  Parent:Recruitment temp. | 0.479  0.010  -0.169  0.119 | 0.051  0.066  0.072  0.094 | 9.379  0.146  -2.345  1.272 | 0.379  -0.120  -0.311  -0.065 | 0.579  0.140  -0.028  0.303 | <0.001  0.88  **0.026**  0.21 |
| **May** |  |  |  |  |  |  |
| Intercept  Parent treatment  Recruitment treatment  Parent:Recruitment temp. | 0.541  -0.025  -0.077  0.033 | 0.045  0.063  0.063  0.089 | 12.088  -0.395  -1.219  0.373 | 0.453  -0.149  -0.201  -0.142 | 0.628  0.100  0.047  0.209 | <0.001  0.69  0.22  0.71 |

**Model**: Recruitment ~ parent treatment * recruitment treatment + (1| tank)

**Table S8**. Cox mixed effects model output for the survival of *Pocillopora acuta* recruits in April and May 2017. Fixed effects were parent temperature, recruitment temperature (and parent: recruitment temperature interaction). Random effects of tile nested within tank was included; 96 tiles within 12 tanks were used each month.

| **Fixed effects** | **Coefficient** | **Coefficient** **(Exponentiated)** | **Coefficient (SE)** | **z** | **P** |
| --- | --- | --- | --- | --- | --- |
| **April** |  |  |  |  |  |
| Parent temp.  Recruitment temp.  Parent:Recruitment temp. | -0.39  -0.11  0.57 | 0.67  0.98  1.76 | 0.22  0.46  0.31 | -1.81  -0.25  1.82 | 0.07  0.80  0.07 |
| **May** |  |  |  |  |  |
| Parent treatment  Recruitment treatment  Parent:Recruitment temp. | -0.26  -0.13  0.88 | 0.77  0.88  2.42 | 0.22  0.36  0.31 | -1.18  -0.37  2.86 | 0.24  0.71  **<0.01** |

**Model**: Survival ~ parent treatment * recruitment treatment + (1| tank) + (1| tile)

**Table S9**. Linear mixed-effect model output for diameter of *Pocillopora acuta* recruit in April and May 2017. Fixed effects were parent temperature, recruitment temperature (and parent: recruitment temperature interaction), and time. A random effect of tile nested within tank was included; 96 tiles within 12 tanks were used each month. The number of individual recruits measured across time points each month were: April: n =1275, and May: n = 1939. Data for week 9 in May was not included due to small sample size.

| **Fixed effects** | **Estimate** | **Standard**  **error** | **t** | **Lower**  **CI** | **Upper**  **CI** | **P** |
| --- | --- | --- | --- | --- | --- | --- |
| **April** |  |  |  |  |  |  |
| Intercept  Parent temp.  Recruitment temp.  Parent:Recruitment temp.  Time | 1.938  -0.163  0.017  -0.004  0.072 | 0.034  0.032  0.047  0.047  0.002 | 57.070  -5.081  0.364  -0.090  35.223 | 1.871  -0.225  -0.076  -0.096  0.068 | 2.004  -0.100  0.110  0.088  0.076 | <0.001  **<0.001**  0.72  0.93  **<0.001** |
| **May** |  |  |  |  |  |  |
| Intercept  Parent treatment  Recruitment treatment  Parent:Recruitment temp.  Time | 2.253  -0.502  -0.039  0.142  0.042 | 0.034  0.035  0.047  0.050  0.003 | 65.389  -14.137  -0.821  2.832  16.005 | 2.185  -0.571  -0.132  0.044  0.037 | 2.320  -0.432  0.054  0.241  0.037 | <0.001  **<0.001**  0.42  **0.006**  <**0.001** |

**Model**: Recruit diameter ~ parent treatment * recruitment treatment + time + (1| tank) + (1| tile)

**Table S10**. Linear mixed-effect model output for Fv/Fm of *Pocillopora acuta* recruits in April and May. Fixed effects were parent temperature, recruitment temperature (and parent: recruitment temperature interaction), and time. A random effect of tile nested within tank was included; 96 tiles within 12 tanks were used each month. The number of individual recruits measured across time points each month were: April: n = 1275, and May: n = 1939. Data for week 9 in May was not included due to small sample size.

| **Fixed effects** | **Estimate** | **Standard**  **error** | **t-value** | **Lower**  **CI** | **Upper**  **CI** | **P-value** |
| --- | --- | --- | --- | --- | --- | --- |
| **April** |  |  |  |  |  |  |
| Intercept  Parent temp.  Recruitment temp.  Parent:Recruitment temp.  Time | 0.733  -0.018  -0.004  -0.007  -0.006 | 0.005  0.004  0.007  0.006  0.0003 | 149.516  -4.229  -0.569  -1.031  -19.971 | 0.724  -0.027  -0.017  -0.019  -0.007 | 0.743  -0.010  0.010  0.006  -0.006 | <0.001  **<0.001**  0.577  0.306  **<0.001** |
| **May** |  |  |  |  |  |  |
| Intercept  Parent treatment  Recruitment treatment  Parent:Recruitment temp.  Time | 0.695  -0.013  -0.004  -0.002  -0.003 | 0.004  0.004  0.005  0.005  0.0004 | 191.323  -3.646  -0.893  -0.420  -9.983 | 0.688  -0.020  -0.014  -0.012  -0.004 | 0.702  -0.006  0.005  0.008  -0.003 | <0.001  **<0.001**  0.38  0.68  **<0.001** |

**Model**: Recruit Fv/Fm ~ parent treatment * recruitment treatment + time + (1| tank) + (1| tile)

**Table S11**: Linear model output of *Pocillopora acuta* colony Fv/Fm held under heated (29.5°C) or control (26°C) conditions as assessed at individual time points; 43, 60, 74 and 90 days at treatment conditions.

| **Fixed effects** | **Estimate** | **Standard**  **error** | **t-value** | **Lower**  **CI** | **Upper**  **CI** | **P** |
| --- | --- | --- | --- | --- | --- | --- |
| **Day 43** |  |  |  |  |  |  |
| Intercept  Parent temp. | 0.724  -0.008 | 0.008  0.011 | 90.97  -0.768 | 0.708  -0.030 | 0.739  0.013 | **<0.001**  0.45 |
| **Day 60** |  |  |  |  |  |  |
| Intercept  Parent temp. | 0.723  -0.042 | 0.010  0.014 | 73.185  -3.077 | 0.704  -0.069 | 0.742  -0.015 | **<0.001**  **0.006** |
| **Day 74** |  |  |  |  |  |  |
| Intercept  Parent temp. | 0.649  -0.060 | 0.017  0.024 | 37.495  -2.470 | 0.615  -0.108 | 0.683  -0.012 | **<0.001**  **0.025** |
| **Day 90** |  |  |  |  |  |  |
| Intercept  Parent temp. | 0.609  0.0002 | 0.014  0.022 | 45.142  0.011 | 0.583  -0.043 | 0.636  0.043 | **<0.001**  0.99 |

**Model for each timepoint**: Colony Fv/Fm ~ treatment

**Table S12**: Linear mixed model output for *Pocillopora acuta* recruit size as assessed at each time point in our study for the recruits settled in April.

| **Fixed effects** | **Estimate** | **Standard**  **error** | **t-value** | **Lower**  **CI** | **Upper**  **CI** | | **P** |
| --- | --- | --- | --- | --- | --- | --- | --- |
| **Week 1** |  |  |  |  |  |  | |
| Intercept  Parent temp.  Recruitment temp.  Parent:Recruitment temp. | 1.867  -0.136  0.061  -0.007 | 0.046  0.047  0.068  0.070 | 40.246  -2.867  0.910  -0.101 | 1.777  -0.228  -0.071  -0.143 | 1.958  -0.429  0.194  0.129 | <0.001  **0.005**  0.37  0.92 | |
| **Week 3** |  |  |  |  |  |  | |
| Intercept  Parent temp.  Recruitment temp.  Parent:Recruitment temp. | 2.301  -1.582  6.380  -2.528 | 0.033  0.028  0.049  0.045 | 69.954  -5.700  1.296  -0.001 | 2.236  -0.213  -0.033  -0.088 | 2.365  -0.104  0.160  0.088 | <0.001  **<0.001**  0.21  0.99 | |
| **Week 7** |  |  |  |  |  |  | |
| Intercept  Parent temp.  Recruitment temp.  Parent:Recruitment temp. | 2.463  -0.167  -0.015  0.054 | 0.034  0.038  0.050  0.057 | 72.058  -4.410  -0.297  0.944 | 2.396  -0.241  -0.113  -0.058 | 2.530  -0.093  0.083  0.165 | <0.001  **<0.001**  0.77  0.35 | |
| **Week 9** |  |  |  |  |  |  | |
| Intercept  Parent temp.  Recruitment temp.  Parent:Recruitment temp. | 2.567  -0.199  -0.054  0.026 | 0.053  0.054  0.074  0.079 | 48.826  -3.692  -0.729  0.329 | 2.464  -0.305  -0.198  -0.129 | 2.970  -0.093  0.091  0.181 | <0.001  **<0.001**  0.46  0.74 | |

**Model for each time point**: Recruit diameter ~ parent treatment * recruitment treatment + (1| tank) + (1| tile)

**Table S13.** Linear mixed model output for *Pocillopora acuta* recruit size as assessed at each time point in our study for the recruits settled in May.

| **Fixed effects** | **Estimate** | **Standard**  **error** | **t-value** | **Lower**  **CI** | **Upper**  **CI** | **P** | |
| --- | --- | --- | --- | --- | --- | --- | --- |
| **Week 1** |  |  |  |  |  | |  |
| Intercept  Parent temp.  Recruitment temp.  Parent:Recruitment temp. | 2.233  -0.513  -0.027  0.171 | 0.036  0.041  0.051  0.058 | 62.247  -12.647  -0.524  2.953 | 2.162  -0.592  -0.127  0.057 | 2.303  -0.433  0.073  0.283 | | <0.001  **<0.001**  0.61  0.004 |
| **Week 3** |  |  |  |  |  | |  |
| Intercept  Parent temp.  Recruitment temp.  Parent:Recruitment temp. | 2.477  -0.506  -0.050  0.110 | 0.041  0.042  0.058  0.060 | 60.141  -12.172  -0.857  1.842 | 2.396  -0.587  -0.165  -0.007 | 2.558  -0.425  0.064  0.228 | | <0.001  **<0.001**  0.40  0.07 |
| **Week 7** |  |  |  |  |  | |  |
| Intercept  Parent temp.  Recruitment temp.  Parent:Recruitment temp. | 2.481  -0.482  -0.010  0.097 | 0.035  0.040  0.049  0.060 | 70.952  -12.007  -0.205  1.624 | 0.069  0.079  0.096  0.117 | 2.550  -0.403  0.086  0.215 | | <0.001  **<0.001**  0.84  0.11 |

**Model for each time point**: Recruit diameter ~ parent treatment * recruitment treatment + (1| tank) + (1| tile)

**Table S14.** Linear mixed model output for *Pocillopora acuta* recruit Fv/Fm as assessed at each time point in our study for the recruits settled in April.

| **Fixed effects** | **Estimate** | **Standard**  **error** | **t-value** | **Lower**  **CI** | **Upper**  **CI** | | **P** |
| --- | --- | --- | --- | --- | --- | --- | --- |
| **Week 1** |  |  |  |  |  |  | |
| Intercept  Parent temp.  Recruitment temp.  Parent:Recruitment temp. | 0.717  -0.011  0.011  -0.020 | 0.008  0.006  0.011  0.009 | 14.161  58.888  15.944  69.028 | 0.703  -0.023  -0.010  -0.038 | 0.732  0.0007  0.033  -0.002 | <0.001  0.07  0.33  **0.03** | |
| **Week 3** |  |  |  |  |  |  | |
| Intercept  Parent temp.  Recruitment temp.  Parent:Recruitment temp. | 0.719  -0.011  -0.020  -0.004 | 0.007  0.010  0.011  0.016 | 87.300  -1.038  -1.805  -0.266 | 0.704  -0.032  -0.042  -0.035 | 0.735  0.010  0.002  0.027 | <0.001  0.30  0.07  0.79 | |
| **Week 7** |  |  |  |  |  |  | |
| Intercept  Parent temp.  Recruitment temp.  Parent:Recruitment temp. | 0.681  -0.011  0.001  -0.004 | 0.008  0.008  0.012  0.011 | 84.637  -1.377  0.081  -0.325 | 0.666  -0.026  -0.022  -0.026 | 0.671  0.005  0.024  0.019 | <0.001  0.17  0.94  0.75 | |
| **Week 9** |  |  |  |  |  |  | |
| Intercept  Parent temp.  Recruitment temp.  Parent:Recruitment temp. | 0.699  -0.055  -0.118  0.010 | 0.008  0.009  0.012  0.013 | 83.344  -6.056  -1.017  0.768 | 0.682  -0.073  -0.034  -0.415 | 0.715  -0.037  0.011  0.035 | <0.001  **<0.001**  0.32  0.77 | |

**Model for each time point**: Recruit Fv/Fm~ parent treatment * recruitment treatment + (1| tank) + (1| tile)

**Table S15.** Linear mixed model output for *Pocillopora acuta* recruit Fv/Fm as assessed at each time point in our study for the recruits settled in May.

| **Fixed effects** | **Estimate** | **Standard**  **error** | **t-value** | **Lower**  **CI** | **Upper**  **CI** | | **P** |
| --- | --- | --- | --- | --- | --- | --- | --- |
| **Week 1** |  |  |  |  |  |  | |
| Intercept  Parent temp.  Recruitment temp.  Parent:Recruitment temp. | 0.675  0.013  -0.006  -0.012 | 0.006  0.006  0.008  0.009 | 116.806  2.083  -0.771  -1.390 | 0.664  0.001  -0.022  -0.029 | 0.686  0.024  0.010  0.005 | **<0.001**  **0.04**  0.45  0.17 | |
| **Week 3** |  |  |  |  |  |  | |
| Intercept  Parent temp.  Recruitment temp.  Parent:Recruitment temp. | 0.700  -0.024  -0.006  0.003 | 0.005  0.006  0.007  0.009 | 151.202  -3.893  -0.886  0.286 | 0.691  -0.036  -0.017  -0.015 | 0.709  -0.012  0.007  0.020 | **<0.001**  **<0.001**  0.38  0.78 | |
| **Week 7** |  |  |  |  |  |  | |
| Intercept  Parent temp.  Recruitment temp.  Parent:Recruitment temp. | 0.647  -0.043  0.001  0.006 | 0.005  0.007  0.007  0.010 | 127.544  -5.970  0.090  0.621 | 0.664 0.057  -0.014  -0.014 | 0.685  -0.029  0.015  0.027 | **<0.001**  **<0.001**  0.93  0.54 | |

**Model for each time point**: Recruit Fv/Fm ~ parent treatment * recruitment treatment + (1| tank) + (1| tile)
